# On the Influence of Syphilis in Pregnant Women

**Published:** 1875-08

**Authors:** 


On the Influence of Syphilis in Pregnant Women, under various modes of Treatment.—Dr. F. Weber, of St. Petersburg, has given the results of his observations in 129 pregnant women suffering from syphilis admitted into the Obuchow Hospital during the ten years 1863–73. Of these patients, 35 were treated only locally or not at all; 35 were submitted to treatment by inunction; in 23, inunction was combined with the external use of iodine (iodide of potassium with tincture of iodine); 19 were treated by the internal use of a combination of iodide of potassium and corrosive sublimate; and in 17 cases iodide of potassium was the only remedy used. He gives abundant statistical details, and sums up as follows: In general, the course of pregnancy was interrupted in 25, or 20 per cent. of the cases; this proportion, however, may be reduced, when it is remembered that of the patients four had erysipelas of the head, one recurrent fever, and one exanthematous typhus. 2. Every method of treatment which interferes with the digestive system predisposes to untimely birth. 3. In the cases submitted to simple local treatment, there were 20 per cent. of premature births; in three, however (suffering from typhus and recurrent fevers, and from extensive formation of abscesses), violent fever appears to have been in part the cause of the untimely labor. 4. In pregnant women who were treated by inunction together with local remedies, there was no disturbance of the course of pregnancy. This confirms Professor Sigmund's conjecture, that the inunction treatment has no injurious influence on the course of pregnancy. 5. In women in whom inunction was either accompanied or followed by the internal use of iodine, the percentage of premature births was 37; this, however, may be reduced to 20 by deducting two severe cases of erysipelas of the head. 6. General treatment with a solution of iodide of potassium and perchloride of mercury was attended by 15 per cent. of premature births. 7. In cases treated by iodide of potassium, 42 per cent. of untimely births occurred. 8. The injurious action of general treatment did not in any way correspond to its duration, but much rather to its effects on the digestive organs. Hence general treatment should be interrupted on the first indication of indigestion in a pregnant woman. 9. The period of pregnancy at which general treatment is commenced appears to have no influence on the occurrence of premature labor. 10. The stage of development of the syphilis seems to be not without influence on the occurrence of untimely birth. 11. The puerperal period ran an abnormal course in 4 out of 14 cases treated locally, in 3 out of 8 treated by inunction and iodine, in 3 out of 4 treated by iodine and sublimate (one of these patients died), and in 4 out of 10 treated by iodide of potassium.—*American Journal Medical Sciences.*

